# Evaluating the effect of LPS from periodontal pathogenic bacteria on the expression of senescence‐related genes in human dental pulp stem cells

**DOI:** 10.1111/jcmm.17594

**Published:** 2022-10-19

**Authors:** Mandana Sattari, Mina Masoudnia, Kazem Mashayekhi, Seyed Mahmoud Hashemi, Nikoo Khannazer, Sepanta Sattari, Saeed Mohammadian Haftcheshmeh, Amir Abbas Momtazi‐Borojeni

**Affiliations:** ^1^ Department of Immunology, Faculty of Medicine Shahid Beheshti University of Medical Sciences Tehran Iran; ^2^ Immunology of Infectious Diseases Research Center Research Institute of Basic Medical Sciences, Rafsanjan University of Medical Sciences Rafsanjan Iran; ^3^ Noncommunicable Diseases Research Center Neyshabur University of Medical Sciences Neyshabur Iran

**Keywords:** CDKN1A, CDKN2A, DPSCs, LPS, senescence, SIRT1, TP53

## Abstract

The human dental pulp stem cells (hDPSCs) are one of the readily available sources of multipotent mesenchymal stem cells (MSCs) and can be considered as a type of tool cells for cell‐based therapies. However, the main limitation in the clinical use of these cells is DPSC senescence, which can be induced by lipopolysaccharide (LPS) of oral pathogenic bacteria. Up to now, far little attention has been paid to exploring the molecular mechanisms of senescence in DPSCs. So, the current study aimed to investigate the underlying molecular mechanism of senescence in hDPSCs stimulated with *Porphyromonas gingivalis (P. gingivalis*) and *Escherichia coli (E. coli)*‐derived LPSs, by evaluating both mRNA and protein expression of four important senescence‐related genes, including TP53, CDKN1A, CDKN2A and SIRT1. To this purpose, hDPSCs were stimulated with different LPSs for 6, 24 and 48 h and then the gene expression was evaluated using quantitative real‐time polymerase chain reaction (qPCR) and western blotting. Following stimulation with *P. gingivalis* and *E. coli*‐derived LPSs, the relative mRNA and protein expression of all genes were significantly up‐regulated in a time‐dependent manner, as compared with unstimulated hDPSCs. Moreover, the hDPSCs stimulated with *P. gingivalis* LPS for 6 and 24 h had the highest mRNA expression of CDKN1A and SIRT1, respectively (*p* < 0.0001), whereas the highest mRNA expression of CDKN2A and TP53 was seen in hDPSCs stimulated with *E. coli* LPS for 48 h (*p* < 0.0001). In summary, because DPSCs have been reported to have therapeutic potential for several cell‐based therapies, targeting molecular mechanisms aiming at preventing DPSC senescence could be considered a valuable strategy.

## 1 INTRODUCTION

Human dental pulp stem cells (hDPSCs) are one of the readily available sources of multipotent mesenchymal stem cells (MSCs), characterized by the clonogenic, plastic‐adherent, highly proliferative cells capable of self‐renewal and differentiation potential into several cell lineages, such as osteo/odontogenic, adipogenic, chondrogenic, myogenic and neurogenic lineages.^1^, ^2^ The hDPSCs express cell surface markers specific for MSCs, such as CD105, CD73 and CD90. However, these cells are negative for surface expression of haematopoietic molecules, including CD14, CD34 and CD45.^1^, ^3^

In recent years, there has been an increasing interest in the use of hDPSCs in cell‐based therapies, as these cells have important features including ease to access, cost–benefit, low immunogenicity, simplicity and convenience of isolation and minimum ethical issues.^4^, ^5^, ^6^ However, before clinical usage of hDPSCs in cell‐based therapies, there is an urgent need to address the biological properties of these cells in response to intrinsic and extrinsic stimuli. The dental pulp tissues, which are the source of DPSCs, are often repeatedly encountered with the various types of stimuli, especially infectious agents, such as Gram‐negative bacteria. In this regard, one of the main stimulators that binds to the toll‐like receptor 4 (TLR4) on the surface of DPSCs and exerts its immunopathological effects is LPS. LPS (a major component of G^−^ bacteria) plays a crucial role in the induction and promotion of inflammation in the oral cavity by inducing the production of several pro‐inflammatory mediators such as tumour necrosis factor‐α (TNF‐α), interleukin‐1 (IL‐1), IL‐6 and CCL8.^7^, ^8^

Increasing research evidence has indicated that inflammatory responses can induce senescence (aging) in MSCs.^9^, ^10^, ^11^, ^12^ In this respect, recent studies by Feng et al. have revealed that stimulation with LPS, to imitate an inflammatory microenvironment, promotes senescence of DPSCs.^13^, ^14^ Although senescence plays physiological roles in the human body, this phenomenon can exert pathological roles in MSCs, which is characterized by the reduced capacity of proliferation and differentiation, and functional disorders.^15^, ^16^ Therefore, the senescence of DPSCs is one of the main challenges faced by therapies targeting tissue regeneration. Up to now, few studies have investigated the molecular mechanism of DPSC senescence induced by LPS. Hence, the current study aimed to investigate the underlying molecular mechanism of senescence in LPS‐stimulated DPSCs by evaluating the expression of four important genes (CDKN1A, CDKN2A, TP53 and SIRT1), which are involved in the process of aging, at the mRNA and protein levels.^17^, ^18^, ^19^, ^20^, ^21^ To this purpose, the current study evaluates and compares the impact of LPS derived from *P. gingivalis* and *E. coli* on hDPSCs.

## 2 MATERIALS AND METHODS

### 2.1 Cell cultures

During routine extraction at Surgery Clinic of Dental School of Shahid Beheshti University of Medical Sciences, normal human impacted third molars were obtained from six healthy subjects, 18–25 years of age after they gave informed consent. In the current study, exclusion criteria for the subjects were as follows: inflammation in the upper mucosa of the impacted tooth, any underlying and systemic disease and wisdom tooth decay. All procedures of the current study were approved by the Ethics Committee of Shahid Beheshti University of Medical Sciences (Sbmu) (Ethics code: IR.SBMU.MSP.REC.1397.150).

The hDPSCs were isolated from impacted third molars by disinfecting the tooth surface using 70% ethanol, cutting around the cementoenamel junction using sterilized dental fissure and then removing coronal pulpal tissue using sterile dental excavator burs. Then, the digestion of the minced dental pulp tissues was performed for 1 h at 37°C using a solution of 3 mg/ml collagenase type I (Sigma‐Aldrich, St. Louis, MO, USA). Then, digested dental pulp tissues were filtered using a 70‐μm cell strainer and centrifuged at 12000 RPM for 5 min. Finally, cell pellets were resuspended in Dulbecco's Modified Eagle's Medium (DMEM)/Ham's F12 (Biosera, England) medium containing 12% fetal bovine serum (FBS) (Gibco, UK), 100 U/ml penicillin and 100 μg/ml streptomycin (Biosera, England) and cultured at 37°C in an incubator containing 5% CO_2_. To obtain precise analysis, all tests were done using the third passage of cells (passage 3).

### 2.2 Cell number determination

The hDPSCs at a density of 0.5 × 10^4^ cells/well were seeded into 24‐well plates in triplicate. The hDPSCs were collected after plating and dissociated and the total cell numbers were counted as follows:

$$\begin{array}{c} \begin{array}{cc} \text{Total number of cells}\;= & \;\text{average number of cells}\times \\ & \;10,000\times \text{dilution coefficient of cell suspension}. \end{array} \end{array}$$

### 2.3 Flow cytometry analysis

To examine the surface markers of hDPSCs, flow cytometry (FCM) was carried out using FACSCalibur flow cytometer (BD FACSCalibur™, BD Biosciences). Briefly, hDPSCs were cultured and propagated up to passage 3. Then detached cells were washed with cold phosphate‐buffered saline (PBS) containing 1% FBS (staining buffer) twice and centrifuged for 5 min at 1200 rpm. Then, hDPSCs were labelled with each fluorochrome‐conjugated specific antibody (CD14, CD34, CD45, CD73, CD90 and CD105) and incubated in the dark place for 10–20 min. In the end, DPSCs were resuspended in staining buffer for FCM analysis using FACSCalibur flow cytometer. Finally, the expression level of each cell surface marker was analysed by measuring the mean fluorescent intensity (MFI), using FlowJo software (Tree Star).

### 2.4 Differentiation assays

To evaluate the ability of hDPSCs to differentiate into osteogenic and adipogenic cells, third passage cells were used. Briefly, hDPSCs at the density of 3 × 10^4^ cells/well were seeded into 24‐well plates and incubated overnight. Then, culture media was replaced with 1 ml complete medium containing DMEM low glucose, FBS 10%, Pen/Strep 1%, 10 mM β_Glycerol phosphate (Sigma), 50 μg/ml ascorbic acid (Sigma) and 10 nM dexamethasone and refreshed every 3 days. 21 days after the initiation of the bone differentiation process, hDPSCs were stained with Alizarin red S (Sigma) to assess extracellular matrix calcification.

For adipogenic differentiation, hDPSCs at the density of 3 × 10^4^ cells/well in complete medium supplemented with DMEM low glucose, FBS 10%, Pen/Strep 1%, 100 mM indomethacin (Sigma), 250 mM dexamethasone, 5 mM insulin and 0.5 mM 3‐isobutyl‐1‐methylxanthine were cultured for 3 weeks. During this period, the culture medium was refreshed every 3 days. After induction, DPSCs were fixed with 4% paraformaldehyde (PFA) and stained with 500 μl Oil Red O (Sigma) to assess the formation of neutral lipid droplets.

### 2.5 LPS stimulation

The hDPSCs at the density of 3 × 10^4^ cells/well were seeded into 24‐well plates and incubated overnight. After incubation, the culture medium received either 1 μg/ml *P. gingivalis* LPS (InvivoGen), 1 μg/ml *E. coli* LPS (Sigma), or normal saline (as a control) once for 6, 24 and 48 h.

### 2.6 RNA extraction cDNA synthesis

After 6, 24 and 48 h of stimulation with different LPSs, the total RNA was manually extracted from DPSCs, using acid guanidinium thiocyanate‐phenol‐chloroform (AGPC) and RNX‐PLUS solution (Sinaclon). Then, the concentration and purity of the isolated RNA were measured using a spectrophotometer (Thermo Fisher Scientific). The synthesis of complementary deoxyribonucleic acid (cDNA) was done using the cDNA Synthesis Kit (Yektatajhiz), according to the manufacturer's protocol. Samples were kept at −20°C until the evaluation of gene expression.

### 2.7 Quantitative real‐time PCR

Specific primers (forward and reverse primers) for GAPDH (as a housekeeping gene), TP53, CDKN1A, CDKN2A and SIRT1 were designed using AlleleID® Software (version 7.0, Premier Biosoft International) (Table 1). After designing, the sequences of all primers were checked and blasted for length, primer dimer, annealing temperature and the possibility of hairpin formation with the entire human genome, using the NCBI site. To measure the mRNA expression of all genes, qPCR using SYBR Green master mix (Amplicon, RealQ Plus 2x Master Mix Green) was performed, according to the manufacturer's protocols, in the Corbett Rotor gene 6000 (QIAGEN) system. The final reaction volume was 20 μl containing 10 μl amplicon master mix, 0.5 μl of each primer (10 μM), 1 μl cDNA and 8 μl DEPC water. The qPCR cycling profile was as follows: 15 min at 95°C, then 40 cycles of 15 s at 95°C (denaturation), 45 seconds at 95°C (annealing) and 10 s 72°C (extension), which was followed by a final melting curve analysis at 65–92°C for 15 seconds. The relative expression of target genes was normalized to the GAPDH gene, internal control and then measured using the 2^−Δct^ method.^22^, ^23^ All experiments were done three times.

**TABLE 1:** Primer sequences

Genes		Sequence (5′‐3′)
GAPDH	Forward primer	*CCTGCACCACCAACTGCTTA*
	Reverse primer	*GGCCATCCACAGTCTTCTGG*
TP53	Forward primer	*GAGCTGAATGAGGCCTTGGA*
	Reverse primer	*CTGAGTCAGGCCCTTCTGTCTT*
CDKN1A	Forward primer	*GGCAGACCAGCATGACAGATT*
	Reverse primer	*GCGGATTAGGGCTTCCTCTT*
CDKN2A	Forward primer	*GAAGGTCCCTCAGACATCC*
	Reverse primer	*TCGGTGACTGATGATCTAAGTT*
SIRT1	Forward primer	*GGCGGCTTGATGGTAATC*
	Reverse primer	*CCACAAGAACTAGAGGATAAGA*

### 2.8 Western blotting analysis

LPS‐treated and untreated cells were lysed with RIPA buffer (Radio immunoprecipitation assay buffer, KPG Co., Kerman, Iran), according to the manufacturer's instruction and then centrifuged at 6000 RPM for 20 minutes. To adjustment of protein analysis through Western blotting, the concentration of total protein was measured by bicinchoninic acid (BCA) protein assay kit (Parstous Co.), according to the manufacturer's instruction. About 30 μg/lane of supernatant were carried out in the presence of sodium dodecyl sulphate (SDS) on 12.5% polyacrylamide gel along with low molecular weight protein marker (Parstous Co.), using Bio‐Rad gel electrophoresis system (Bio‐Rad, Hercules, CA). Then, the separated proteins were transferred onto the polyvinylidene difluoride (PVDF) membrane by electroblotting and blocked with PBS buffer containing 2% bovine serum albumin (BSA), 1% polyvinylpyrrolidone (PVP) at 4°C overnight. After washing with PBS buffer, each membrane was incubated with primary antibodies (1:500–1:10000 diluted in 1% BSA) along with anti‐human GAPHD antibody (1:500–1:2500 diluted in 1% BSA) as a reference protein for 24 h at 4°C. Subsequently, sheets were washed with PBS‐Tween buffer and incubated with goat anti‐rabbit HRP‐conjugated secondary antibody (1:500–1:20000 diluted in 1% BSA) for 2 h at room temperature and then washed with PBS‐Tween buffer. Specific bands were detected by chemiluminescent substrate (Parstous), according to the manufacturer's instruction with Bio‐Rad ChemiDoc XRS System. The following antibodies were used: rabbit anti‐human GAPDH (Abcam), Rabbit anti‐human TP53 (Sigma‐Aldrich), Rabbit anti‐human SIRT1 (Abcam), Rabbit anti‐human CDKN1A (Thermofisher), Rabbit anti‐human CDKN2A (Abcam) and goat anti‐rabbit HRP‐conjugated secondary antibody (Abcam). Finally, the mean intensity of the immunoblot sheets was calculated by the semi‐quantitative analysis of each blot area in triplicates, using ImageJ software Version 1.52a [National Institutes of Health (NIH)].

### 2.9 Statistical analysis

Data analysis was performed using GraphPad Prism software (Version 8.0.2, San Diego, California). Comparisons between groups were performed using one‐way analysis of variance (anova) followed by Tukey's multiple comparison tests. Descriptive data were generated for all variables. Significance levels were set at <5%.

## 3 RESULTS

### 3.1 Characterization of hDPSCs

**FIGURE 1:**
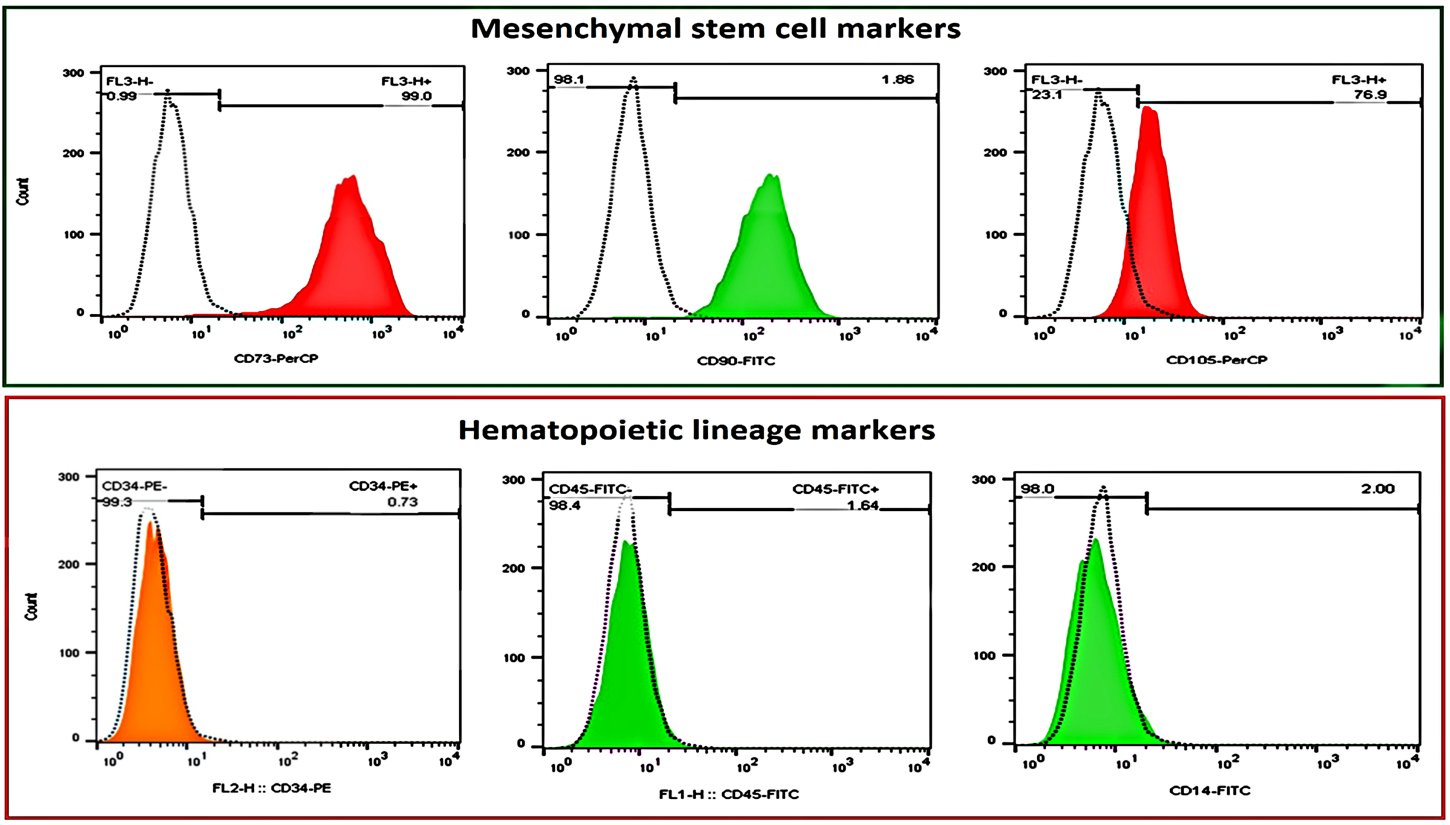
FCM analysis of cell surface markers including CD73, CD90, CD105, CD34, CD45f and CD14. The hDPSCs were cultured and propagated up to passage 3. Then, the MFI of cell surface markers was calculated using FCM analysis. The hDPSCs expressed high levels of MSC markers including CD73 (99.0 ± 4.2), CD90 (98.1 ± 7.3) and CD105 (76.9 ± 3.7). In contrast, these cells expressed low levels of haematopoietic lineage markers including CD34 (0.73 ± 0.08), CD45 (1.64 ± 0.2), and CD14 (2.0 ± 0.3). The results are expressed as mean ± SD (*n* = 3).

To better identify the characterization of hDPSCs, the MFI of cell surface markers was calculated by FCM analysis and results have been shown in Figure 1. Analysis of surface marker profile clearly indicated that hDPSCs expressed high levels of mesenchymal stem cell markers including CD73 (99.0 ± 4.2), CD90 (98.1 ± 7.3) and CD105 (76.9 ± 3.7). In contrast, these cells expressed low levels of haematopoietic lineage markers including CD34 (0.73 ± 0.08), CD45 (1.64 ± 0.2) and CD14 (2.0 ± 0.3) (Figure 1).

### 3.2 Differentiation of hDPSCs

**FIGURE 2:**
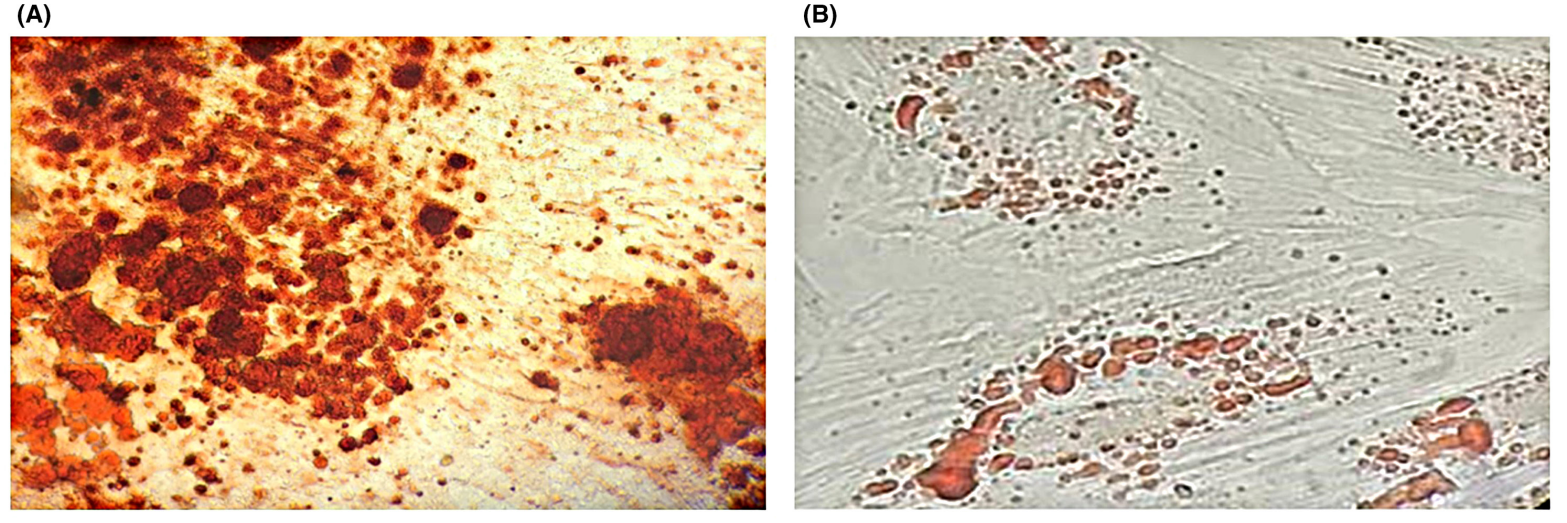
Differentiation assay. (A) Undifferentiated hDPSCs as the control had no calcium and lipid accumulations. (B) After 21 days of differentiation in osteo/odontogenic inductive medium, hDPSCs differentiated into the osteogenic lineage, as confirmed by the presence of extracellular calcium nodules (red areas). (C) Under adipogenic differentiation process (21 days in adipogenic inductive medium), hDPSCs were able to differentiate into adipogenic lineage, as confirmed by the presence of natural lipid vacuoles (red areas).

As shown in Figure 2B, after 21 days of differentiation in osteo/odontogenic inductive medium, hDPSCs differentiated into the osteogenic lineage, as confirmed by the presence of extracellular calcium nodules (red areas). Moreover, under the adipogenic differentiation process (21 days in adipogenic inductive medium), hDPSCs were able to differentiate into the adipogenic lineage, as confirmed by the presence of natural lipid vacuoles (red areas) (Figure 2C). In contrast, undifferentiated hDPSCs as the control had no calcium and lipid accumulations (Figure 2A).

### 3.3 Expression of CDKN1A, CDKN2A, TP53 and SIRT1

**FIGURE 3:**
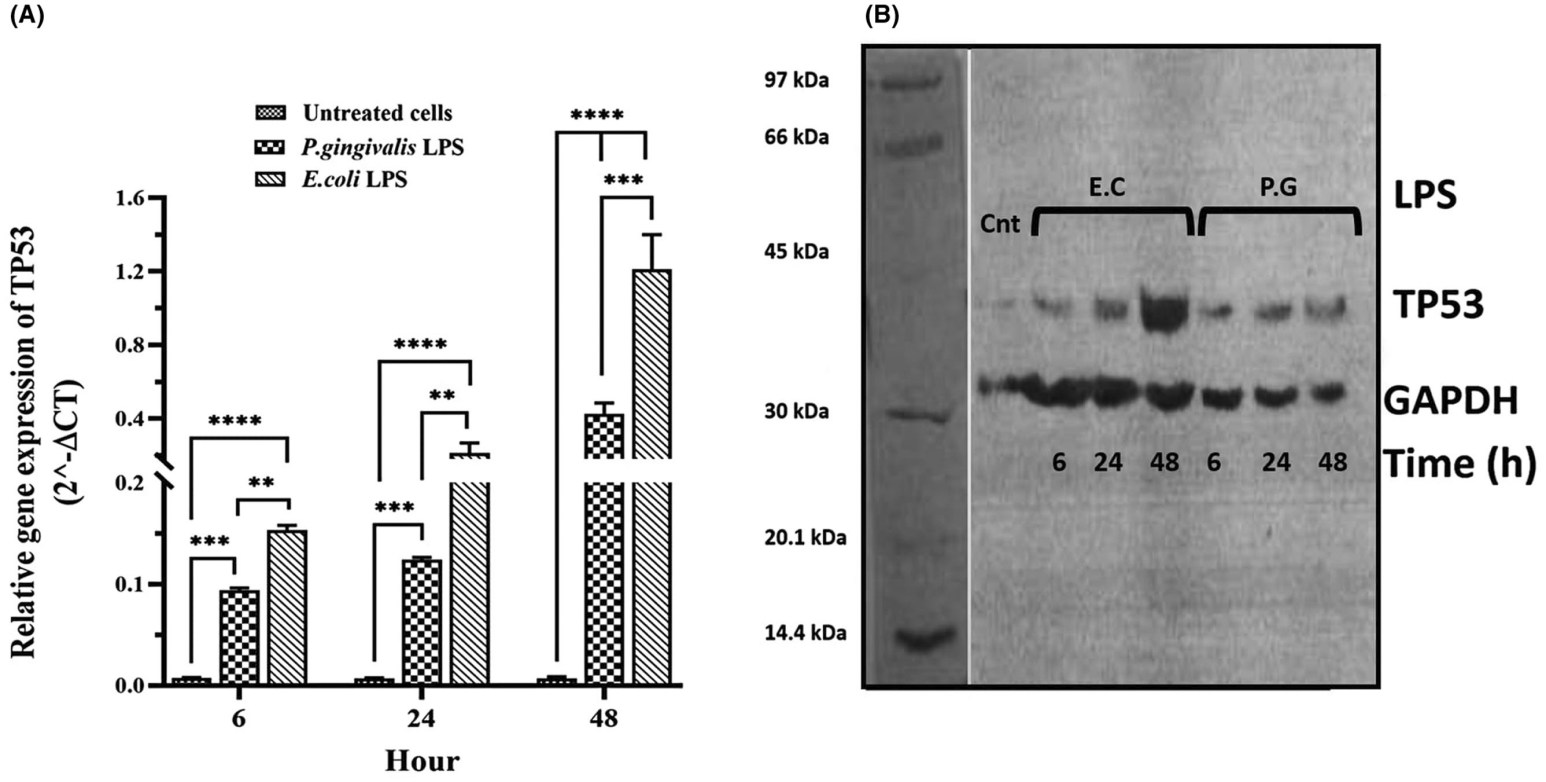
mRNA and protein expression of TP53 gene. The hDPSCs at the density of 3 × 10^4^ cells/well were seeded into 24‐well plates and then treated with either 1 μg/ml *P. gingivalis* LPS, 1 μg/ml *Escherichia coli* LPS, or normal saline (as control or untreated) once for 6, 24 and 48 h. Both mRNA (A) and protein levels (B) of TP53 significantly up‐regulated following treatment with different LPSs, in a time‐dependent manner, as compared to unstimulated hDPSCs. In the part B, the first column shows molecular weight markers. The results are expressed as mean ± SD (*n* = 3). TP53: 44 kDa, GAPDH: 37 kDa. Cnt: Control, P. G: *P. gingivalis*, E.C*: E. coli*. **p < 0.01, ***p < 0.001, ****p < 0.0001.

**FIGURE 4:**
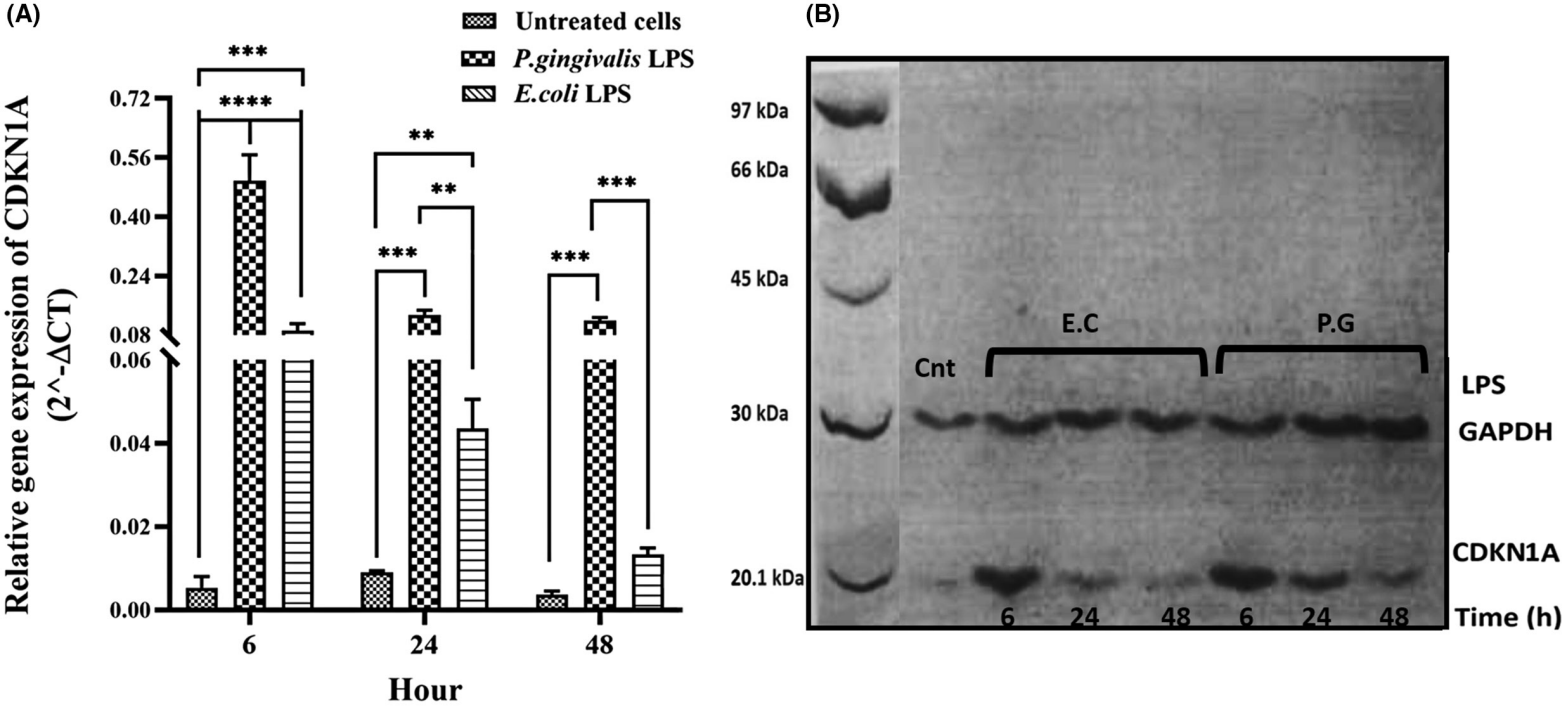
mRNA and protein expression of CDKN1A gene. The hDPSCs at the density of 3 × 10^4^ cells/well were seeded into 24‐well plates and then treated with either 1 μg/ml *Porphyromonas gingivalis* LPS, 1 μg/ml *Escherichia coli* LPS, or normal saline (as control or untreated) once for 6, 24 and 48 h. Both mRNA (A) and protein levels (B) of CDKN1A significantly up‐regulated following treatment with different LPSs, in a time‐dependent manner, as compared with unstimulated hDPSCs. In the part B, the first column shows molecular weight markers. The results are expressed as mean ± SD (*n* = 3). CDKN1A: 18 kDa, GAPDH: 37 kDa. Cnt: Control, P. G: *P. gingivalis*, E.C*: E. coli*. **p < 0.01, ***p < 0.001, ****p < 0.0001.

**FIGURE 5:**
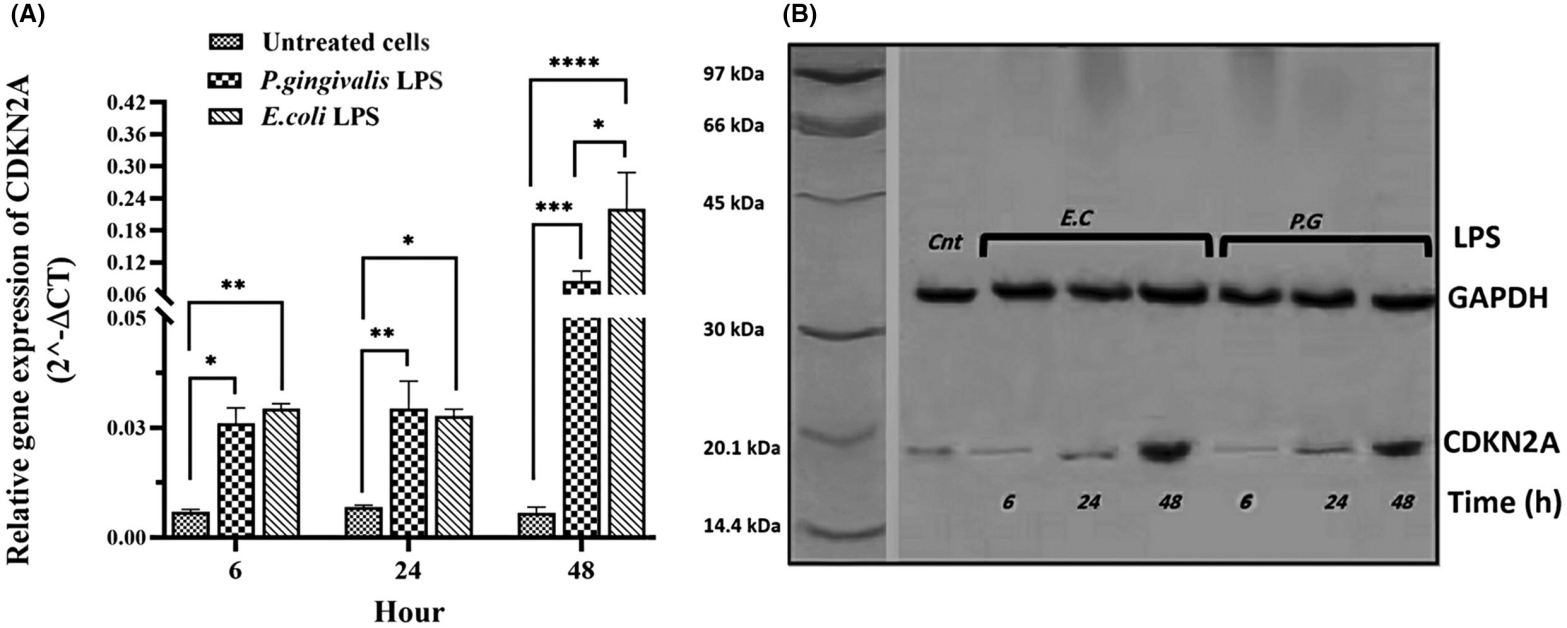
mRNA and protein expression of CDKN2A gene. The hDPSCs at the density of 3 × 10^4^ cells/well were seeded into 24‐well plates and then treated with either 1 μg/ml *Porphyromonas gingivalis* LPS, 1 μg/ml *Escherichia coli* LPS, or normal saline (as a control or untreated) once for 6, 24, and 48 h. Both mRNA (A) and protein levels (B) of CDKN2A significantly up‐regulated following treatment with different LPSs, in a time‐dependent manner, as compared with unstimulated hDPSCs. The results are expressed as mean ± SD (*n* = 3). CDKN2A: 17 kDa, GAPDH: 37 kDa. Cnt: Control, P. G: *P. gingivalis*, E.C*: E. coli*. *p < 0.05, **p < 0.01, ***p < 0.001, ****p < 0.0001.

**FIGURE 6:**
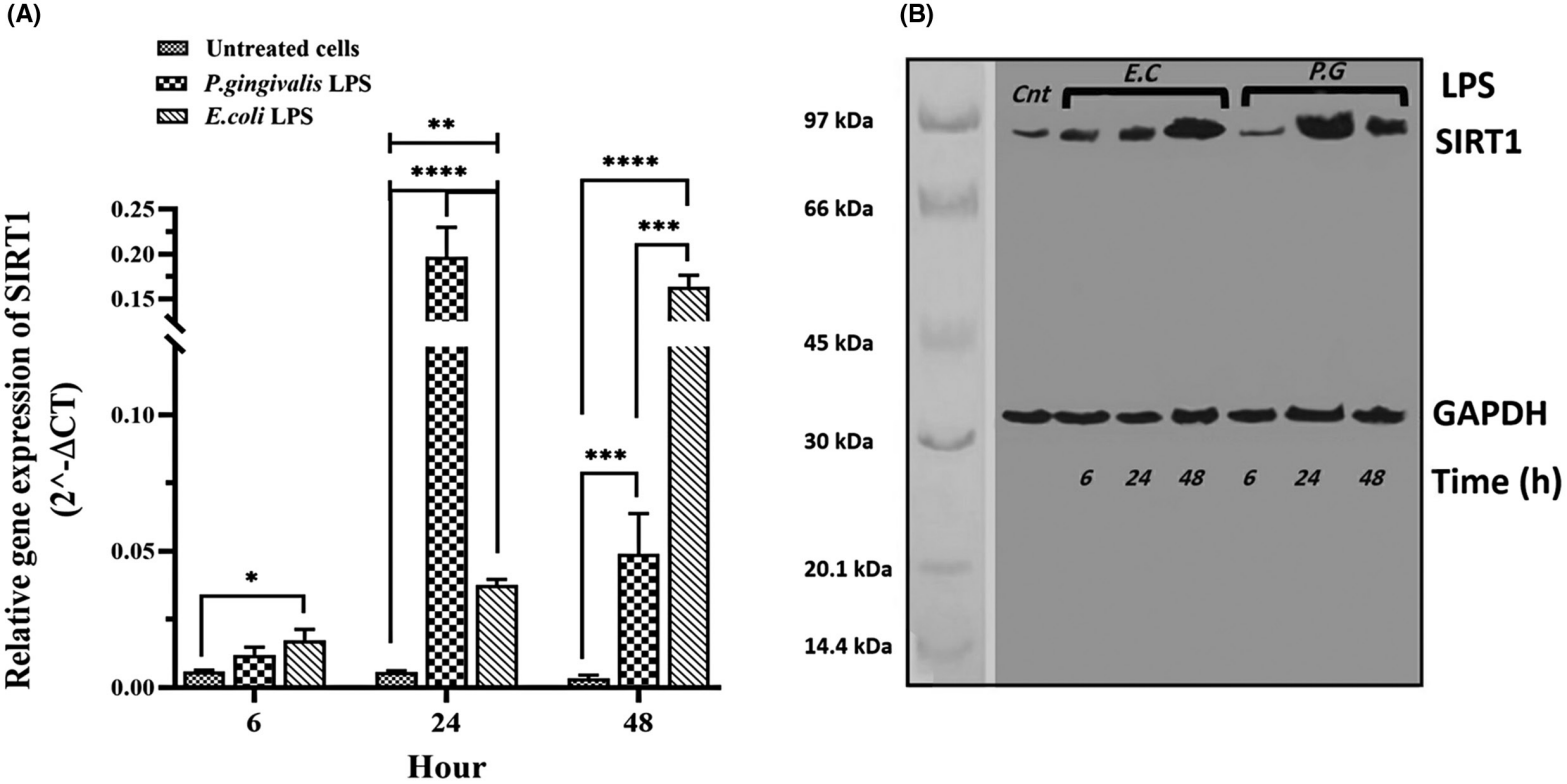
mRNA and protein expression of SIRT1 gene. The hDPSCs at the density of 3 × 10^4^ cells/well were seeded into 24‐well plates and then treated with either 1 μg/ml *Porphyromonas gingivalis* LPS, 1 μg/ml *Escherichia coli* LPS, or normal saline (as a control or untreated) once for 6, 24 and 48 h. Both mRNA (A) and protein levels (B) of SIRT1 significantly up‐regulated following treatment with different LPSs, in a time‐dependent manner, as compared to unstimulated hDPSCs. The results are expressed as mean ± SD (*n* = 3). SIRT1: 81 kDa, GAPDH: 37 kDa. Cnt: Control, P. G: *P. gingivalis*, E.C*: E*. coli. *p < 0.05, **p < 0.01, ***p < 0.001, ****p < 0.0001.

The gene expression of CDKN1A, CDKN2A, TP53 and SIRT1 was evaluated in hDPSCs after 6, 24 and 48 h of stimulation with different LPSs (*P. gingivalis* LPS and *E. coli* LPS) by qPCR and Western blotting, and the results are presented in Figures 3 and 6 and Tables 2 and 3. After stimulation with different LPSs, the relative mRNA expression and protein levels of all genes were significantly up‐regulated in a time‐dependent manner, as compared with unstimulated hDPSCs (Figures 3, 4, 5, 6). Further analysis showed that hDPSCs stimulated with *P. gingivalis* LPS for 6 and 24 h had the highest mRNA level and immunoblot area intensity for CDKN1A (0.49 ± 0.07 and 69.93 ± 11.36, respectively), and SIRT1 (0.19 ± 0.04 and 164.0 ± 21.10, respectively), respectively (Figures 4 and 6; Tables 2 and 3) (*p* < 0.0001), whereas the highest mRNA level and intensity of immunoblot areas for TP53 (1.21 ± 0.26 and 100.4 ± 25.83, respectively), and CDKN2A (0.22 ± 0.09 and 77.35 ± 31.80, respectively) were detected in hDPSCs stimulated with *E. coli* LPS for 48 h (Figures 3 and 5; Tables 2 and 3) (*p* < 0.0001). In terms of exposure time to LPS, there is a trend of increase in the mRNA expression levels and immunoblot intensity of TP53 and CDKN2A in parallel with the increase in the stimulation time with different LPSs [*P. gingivalis* LPS (*p* < 0.01 for two genes) and *E .coli* LPS (*p* < 0.0001 for two genes)] (Figures 3 and 5; Tables 2 and 3), while a trend of decrease in the mRNA expression levels and immunoblot intensity of CDKN1A was observed in parallel with increase in the exposer time with *P. gingivalis* LPS (*p* < 0.0001) and *E .coli* LPS (*p* < 0.05) (Figure 4). The mRNA and immunoblot intensity of SIRT1 were found to be up‐regulated in parallel with the increase in the exposure time with *E. coli* LPS (*p* < 0.0001), while a significant increase in the gene expression of SIRT1 was only seen after 24 h of treatment with *P. gingivalis* LPS (*p* < 0.0001) and this up‐regulation was markedly decreased after 48 h of treatment (*p* < 0.0001) (Figure 6).

**TABLE 2:** Relative mRNA expression of TP53, CDKN1A, CDKN2A and SIRT1 following stimulation of DPSCs with *Porphyromonas gingivalis* and *Escherichia coli*‐derived LPS

Genes	Times	*P. gingivalis* LPS	*E. coli* LPS
		Mean ± SD	Mean ± SD
TP53	6 h	0.07 ± 0.00	0.12 ± 0.00
	24 h	0.10 ± 0.00	0.21 ± 0.07
	48 h	0.42 ± 0.08	1.21 ± 0.26
CDKN1A	6 h	0.49 ± 0.07	0.09 ± 0.01
	24 h	0.13 ± 0.01	0.04 ± 0.00
	48 h	0.11 ± 0.00	0.01 ± 0.00
CDKN2A	6 h	0.026 ± 0.00	0.029 ± 0.00
	24 h	0.029 ± 0.00	0.028 ± 0.00
	48 h	0.086 ± 0.02	0.22 ± 0.09
SIRT1	6 h	0.01 ± 0.00	0.01 ± 0.00
	24 h	0.19 ± 0.04	0.03 ± 0.00
	48 h	0.05 ± 0.02	0.16 ± 0.01

**TABLE 3:** Immunoblot image analysis

Genes	Intensity of immunoblot areas					
	*Porphyromonas gingivalis LPS*			*Escherichia coli LPS*		
	6 h	24 h	48 h	6 h	24 h	48 h
TP53	63.1 ± 14.1	75.3 ± 17.5	93.4 ± 14.5	67.7 ± 8.8	80.2 ± 15.5	100.4 ± 25.8
CDKN1A	69.9 ± 11.3	63.5 ± 9.8	49.8 ± 2.7	67.4 ± 8.3	51.7 ± 2.7	48.7 ± 3.5
CDKN2A	23.6 ± 0.9	34.3 ± 6.8	74.9 ± 18.2	34.7 ± 1.4	33.4 ± 3.7	77.3 ± 31.8
SIRT1	83.5 ± 9.7	164.0 ± 21.1	121.8 ± 25.6	102.3 ± 10.4	110.3 ± 16.7	141.9 ± 23.6

*Note*: The results are expressed as mean ± SD (*n* = 3).

## 4 DISCUSSION

The key question of the current study was whether treatment with LPS could alter the expression levels of TP53, CDKN1A, CDKN2A and SIRT1 in hDPSCs. For the first time, our in vitro experiments found that following stimulation with *P. gingivalis* and *E. coli*‐derived LPSs, the expression of TP53, CDKN1A, CDKN2A and SIRT1 markedly up‐regulated in comparison with unstimulated DPSCs, at a time‐dependent manner.

The dental pulp tissues as the main source of DPSCs are frequently affected by G^−^ bacteria and their pathogenic products. LPS (endotoxin) derived from these bacteria is one of the main stimulators of inflammatory responses in the oral cavity. On the contrary, there is a close relationship between inflammation and senescence. Prior studies indicated that stimulation with LPS by imitating inflammatory conditions may affect the biological properties of DPSCs by inducing cellular senescence. DPSCs express high levels of membrane pattern recognition receptors (PRR) such as toll‐like receptor 4 (TLR‐4), which binds to the LPS of G^−^ negative periodontopathic bacteria in the oral cavity and initiate inflammatory signalling cascades including the activator protein 1 (AP1), nuclear factor‐κB (NF‐κB), mitogen‐activated protein kinases (MAPKs) and myeloid differentiation factor 88 (MyD88) signalling pathways.^24^ As a consequence of these events, several inflammatory responses are promoted, which in turn may stimulate senescence in DPSCs.^13^, ^14^ In this regard, several important genes such as TP53, CDKN1A, CDKN2A and SIRT1 play key regulatory roles in the DPSC senescence.

TP53, a 53‐kDa protein located at position 17p13.1, is one of the first transcription factors that play crucial regulatory roles in several cellular events such as self‐renewal, genome stability, cell‐cycle arrest and apoptosis.^25^, ^26^ Importantly, several triggers such as DNA damage, oxidative stress and inflammation can initiate the p53 signalling cascade, which eventually leads to cellular senescence.^25^, ^26^ In the downstream of p53 signalling pathway, CDKN1A gene as the first identified senescence‐associated target gene of p53 has been located, which encodes the cyclin‐dependent kinase (CDK) inhibitor p21.^25^ Several research reports have revealed that p21 effectively is capable of inducing cellular senescence in vitro.^27^, ^28^, ^29^, ^30^, ^31^ Indeed, p21 has been defined as a pivotal mediator of p53‐regulated cellular senescence in response to various stimuli such as DNA damage and inflammation.^31^, ^32^ In this respect, our in vitro study showed that the exposure of DPSCs with *P. gingivalis* and *E.coli*‐derived LPSs markedly up‐regulates the mRNA and protein expression of TP53 and CDKN1A in hDPSCs. Meanwhile, the changes in the expression levels of TP53 and CDKN1A were dependent on the time of stimulation with LPS, so a clear trend of increasing and decreasing in the mRNA and protein expression of TP53 and CDKN1A was seen with time, respectively. A possible explanation for these results may be the negative feedback loop between the expression of TP53 and CDKN1A, so that increase in the expression of CDKN1A in the downstream of TP53 signalling pathway might be in turn downregulates mRNA and protein expression of p53. Consist with our findings, it has been shown that stimulation with *E. coli* LPS at the concentration of 10 ng/ml introduces senescence of DPSCs by up‐regulating the expression of p53 and p21, in a time‐dependent manner.^14^ In the current study, for the first time, we used the *P. gingivalis*‐derived LPS for investigating the underlying molecular mechanism of DPSC senescence. Evidence suggests that periodontopathic bacteria, especially *P. gingivalis*, via several virulence factors including LPS, peptidoglycans, and lipoteichoic acid, are important inducers of the inflammation in the oral cavity, therefore, could be considered as the important inducer of DPSC senescence.^33^, ^34^, ^35^ In this regard, the current study found that *P. gingivalis*‐derived LPS can markedly up‐regulate the gene expression of TP53 and CDKN1A in hDPSCs. Meanwhile, *P. gingivalis* LPS also has a high capacity to increase the mRNA and protein expression of CDKN1A, over *E. coli* LPS.

One of the most important findings in the current study was that hDPSCs treated with P*. gingivalis* and *E. coli* ‐derived LPSs had a high level of CDKN2A, compared with unstimulated hDPSCs, in a time‐dependent manner. CDKN2A is one of the main regulatory proteins, which encodes by the p16 gene, participating in the G1/S cell cycle checkpoint.^36^ This protein was reported to be a unique marker for cell senescence in vitro and in vivo.^37^ As a result of exposure to several stimuli such as inflammation, CDKN2A gene expression is increased, leading to the stabilization of p53 protein through its inhibitory effects on the E3 ubiquitin‐protein ligase MDM2 (a protein responsible for the degradation of p53).^36^, ^37^, ^38^ Interestingly, Feng et al. found that treatment with *E. coli* LPS by recruiting TLR4 signalling pathway markedly increased P16 expression at the mRNA and protein levels, which eventually leads to the induction of DPSC senescence.^13^ Importantly, p16 short interfering RNA (siRNA) is capable of reversing LPS‐mediated DPSC senescence.^13^ In line with these findings, the current study also indicated the increased gene and protein expression of CDKN2A following treatment with *E. coli* LPS. Moreover, for the first time, our result showed that *P. gingivalis* LPS is efficacious to significantly upregulate the mRNA and protein expression of CDKN2A, in a time‐dependent manner.

One interesting finding in the current study was that following stimulation with *P. gingivalis* and *E. coli*‐derived LPSs, the expression of SIRT1 markedly up‐regulated. In this respect, our results found a different pattern of SIRT1 expression following stimulation with different LPSs, so that *P. gingivalis* LPS had the highest impact on the mRNA expression of SIRT1 and this increased expression of SIRT1 markedly downregulated after 48 h of stimulation. Moreover, a clear trend of increase in the expression of SIRT1 was observed in *E. coli* LPS‐stimulated hDPSCs, in a time‐dependent manner.

The mammalian sirtuins (SIRT) are a family of NAD^+^‐dependent histone deacetylase with homology to the *saccharomyces cerevisiae* silent information regulator 2 (Sir2) and.^19^ SIRT1 plays key regulatory roles in several biological processes including DNA repair, cell cycle regulation, inflammation, apoptosis, aging and autophagy.^19^, ^39^, ^40^ SIRT1 also is an important negative regulator of p53 through deacetylation of this protein in different sites.^41^ There is evidence that SIRT1 by hampering the p53 signalling pathway and its downstream target genes especially CDKN1A is capable of preventing cellular senescence, therefore, has an anti‐senescence activity.^41^ For the first time, our finding indicated that LPS‐stimulated hDPSCs had a high level of SIRT1 expression both at the mRNA and protein levels. A possible explanation for our results may be a compensatory mechanism by which SIRT1 expression has been upregulated parallel to the increased expression of TP53 to inhibit cellular senescence in hDPSCs.

## 5 CONCLUSION

This is the first study that has investigated the impact of *P. gingivalis* and *E. coli*‐derived LPSs on the expression of TP53, CDKN1A, CDKN2A and SIRT1 in hDPSCs and contributes to existing knowledge by providing further evidence on underlying molecular mechanisms of DPSC senescence. Our findings obviously revealed that stimulation with different LPSs markedly upregulated the expression of TP53, CDKN1A, CDKN2A and SIRT1 both at the mRNA and protein levels, in a time‐dependent manner. Because DPSCs are one of the readily available sources of MSCs for several cell‐based therapies, targeting molecular mechanisms aiming at preventing DPSC senescence could be considered has as a valuable strategy. Hereupon, it would be interesting to carry out further research to modulate TP53, CDKN1A, CDKN2A and SIRT1.

## AUTHOR CONTRIBUTIONS

**Mandana Sattari:** Project administration (equal); writing – original draft (equal). **Mina Masoudnia:** Data curation (equal); writing – original draft (equal). **Kazem Mashayekhi:** Investigation (equal). **Seyed Mahmoud Hashemi:** Data curation (equal); formal analysis (equal). **Nikoo Khannazer:** Formal analysis (equal); software (equal). **Sepanta Sattari:** Data curation (equal); software (equal). **Saeed Mohammadian Haftcheshmeh:** Conceptualization (equal); validation (equal). **Amir Abaas Momtazi‐Borojeni:** Writing – review and editing (lead).

## FUNDING INFORMATION

This study was financially supported by Faculty of Medicine, Shahid Beheshti University of Medical Sciences (sbmu) (Grant Number: 971372).

## CONFLICT OF INTEREST

The authors declare that there are no conflicts of interest.

## Data Availability

The data that support the findings of this study are available from the corresponding author upon reasonable request.
